# Acquired Hemophilia—A Case Series and Review

**DOI:** 10.3390/jcm14051597

**Published:** 2025-02-26

**Authors:** Liat Waldman Radinsky, Maayan Sivan, Aharon Lubetsky, Mudi Misgav, Shadan Lalezari, Omri Cohen, Tlalit Barhod, Gili Kenet, Orly Efros

**Affiliations:** 1 National Hemophilia Center, Sheba Medical Center, Ramat-Gan 5266202, Israel; aharon.lubetsky@sheba.health.gov.il (A.L.); mudi.misgav@sheba.health.gov.il (M.M.); shadan.lalezari@sheba.health.gov.il (S.L.); tlalit.barhod@sheba.health.gov.il (T.B.); gili.kenet@sheba.health.gov.il (G.K.); 2 Sheba Medical Center, Ramat-Gan 5266202, Israel; maayan.siv2@gmail.com; 3 Medical School, University of Nicosia, 2408 Nicosia, Cyprus; 4 Faculty of Medical & Health Sciences, Tel-Aviv University, Tel-Aviv 6997801, Israel; 5 Department of Transfusion Medicine, Kaplan Medical Center and Faculty of Medicine, Hebrew University of Jerusalem, Jerusalem 7661041, Israel; omrico@clalit.gov.il; 6 Department of Medicine and Surgery, University of Insubria, 21100 Varese, Italy; 7 Department of Molecular Cell Biology, Weizmann Institute of Science, Rehovot 7610001, Israel

**Keywords:** acquired hemophilia, acquired bleeding disorder, Emicizumab, Rituximab, pregnancy-induced acquired hemophilia

## Abstract

**Background:** Acquired hemophilia A (AHA) is a rare, life-threatening autoimmune disorder characterized by inhibitory autoantibodies against factor VIII (FVIII), resulting in spontaneous or trauma-related bleeding. This study reviews a single-center cohort to evaluate patient characteristics, treatments, and outcomes. **Methods:** We retrospectively reviewed the records of 22 adult patients diagnosed with AHA between 2012 and 2024. The data included demographics, clinical presentation, laboratory findings, treatments, and outcomes. Statistical analysis compared genders and evaluated treatment strategies and remission outcomes. **Results:** The cohort had an equal gender distribution with an average age of 62 years (22–102 years). Suspected etiologies included pregnancy (27%), malignancy (23%), autoimmune diseases (5%), and idiopathic causes (45%). The most common presentation was spontaneous cutaneous hematoma (82%). Severe bleeding necessitating hemostatic therapy occurred in 9% of cases. Initial immunosuppressive therapy (IST) with corticosteroids achieved remission in 45% of patients, while additional treatment with Rituximab or Cyclophosphamide was required in others. Emicizumab, a novel FVIII-mimetic, was successfully used in one patient with severe refractory bleeding. Remission was achieved in 64% of patients within a median of 3 months, with a recurrence rate of 14%. No thrombotic events were observed, although corticosteroid side effects, including one hip fracture, were noted. **Conclusions:** IST remains the cornerstone of AHA treatment, though side effects necessitate individualized care. Emicizumab shows promise, particularly in refractory cases and fragile populations. Future research is needed to evaluate long-term outcomes and spontaneous remission rates, especially in special populations like post-partum women and the elderly.

## 1. Introduction

Acquired hemophilia A (AHA) is a rare autoimmune disorder in which inhibitory autoantibodies develop against clotting factor VIII (FVIII). This results in potentially life-threatening spontaneous bleeding or trauma-induced bleeding [1,2].

While AHA can affect individuals of all ages, it is most prevalent among the elderly, with a median age of onset around 70–76 years. It affects both sexes equally, and is often linked to age-related immune dysfunction and comorbidities [2,3].

The incidence of AHA is estimated to range from 0.65 to 1.5 cases per million people per year. Half of cases are associated with autoimmune diseases, malignancies, infections, or pregnancy, while the remaining cases are idiopathic with no identifiable cause [1,2].

Approximately 10% of AHA cases can be attributed to malignancy [4]. Among them are hematological malignancies in which chronic lymphocytic leukemia (CLL) is predominant, causing up to 30% of all cases. Within solid tumors causing AHA, roughly 50% of cases are attributed to an underlying carcinoma of the prostate or lung carcinoma [4].

Obstetric-associated AHA is more common in the post-partum period, appearing mostly up to 3 months after delivery, but can be diagnosed up to a year post-partum. This condition should be suspected in the presence of unexplained excessive or prolonged vaginal bleeding or large soft-tissue hematomas at multiple sites [5]. Primigravid is thought to be a risk factor for post-partum AHA [5]. It is also important to note that when AHA occurs during pregnancy, maternal IgG antibodies can cross the placenta and, in rare cases, cause bleeding manifestations in the fetus or the newborn [6,7].

AHA associated with autoimmune disease has also been described with illnesses such as rheumatoid arthritis, systemic lupus erythematosus (SLE), Sjogren’s syndrome, and dermatomyositis [1].

Several cases of AHA that were described as drug-induced have been reported [8]. clinical symptoms of AHA often include bleeding into soft tissues—mostly subcutaneous and intramuscular bleeds, which are often spontaneous. More severe bleeding manifestations can also occur at presentation and include intracranial hemorrhage, bleeding of the genitourinary system, and gastrointestinal bleeding [1]. These clinical manifestations are different from the clinical presentation of the congenital form of hemophilia, which is characterized by spontaneous bleeding into joints and excessive bleeding after trauma, injury, or invasive interventions.

Diagnosis of AHA is often delayed due to its rarity, nonspecific symptoms, and a lack of awareness among medical professionals. Atypical bleeding symptoms, along with prolonged activated partial thromboplastin time (aPTT) that does not correct with a mixing study, is the hallmark diagnostic clue, accompanied by reduced FVIII activity and the presence of inhibitors, confirmed by a Bethesda assay [3].

Treatment aims to achieve two primary goals: controlling bleeding and eradicating the inhibitor. Hemostatic management typically involves bypassing agents such as recombinant activated factor VII (rFVIIa) or activated prothrombin complex concentrate (aPCC) [1,3], sometimes with the support of tranexamic acid. Bleeding can also be controlled with recombinant FVIII (rFVIII). Immunosuppressive therapy (IST), including corticosteroids, Rituximab, or Cyclophosphamide, is used to suppress autoantibody production [1,3]. In past years, treatment with IVIg was considered optional, but current guidelines discourage this practice [3].

To note, in recent years, Emicizumab, a bispecific monoclonal antibody that mimics the action of FVIII, approved for congenital hemophilia A with and without FVIII inhibitors, has emerged as a novel off-label therapeutic option for treating AHA [9,10]. Emicizumab mimics FVIII functions by bridging activated factor IX and factor X, effectively bypassing the need for FVIII. Early studies and case reports suggest that Emicizumab can provide effective prophylaxis and reduce bleeding episodes, though further research is needed to fully assess its long-term efficacy and safety in AHA patients [9,10,11].

In this study, we aim to review a cohort of patients with AHA followed at a large tertiary referral center and assess their course, treatments, and outcomes. Our study also reviews and addresses the current recommendations for the treatment of AHA.

## 2. Methods

We conducted a retrospective study of all adult patients (age > 18) diagnosed with AHA at the Sheba Medical Center between the years 2012 and 2024.

The inclusion criteria were as follows: (1) age > 18, (2) a positive level (>0.5 BU) of antibodies against FVIII (3) continued ambulatory follow-up at our coagulation clinic. The exclusion criteria included (1) patients with concomitant congenital coagulopathis (congenital hemophilia A, or other rare bleeding disorders), and (2) patients diagnosed during hospitalization who were never followed in our ambulatory coagulation clinic.

Retrospective patient data, which were prospectively collected during hospitalization and follow-up, were retrieved using the MDClone platform, a secure system designed to generate data from the hospital’s digital health records while preserving the privacy of individuals and ensuring compliance with ethical standards.

Notably, all laboratory assays were conducted in our specialized coagulation laboratory. FVIII was determined using one-stage assays and standard techniques, FVIII inhibitors were assayed using Bethesda units (BUs), and for Emicizumab-treated patients, chromogenic assays using bovine reagents were performed as required [11].

Eligible patient records were manually reviewed to extract the following information:

- Demographics: age, sex, and other relevant details.
- Medical History: past medical conditions and comorbidities.
- AHA Presentation: clinical symptoms and initial presentation.
- Laboratory Results: including aPTT, FVIII activity levels, and FVIII inhibitor levels.
- Clinical Outcomes: recurrence of bleeding episodes and mortality.

Data analysis included comparisons of means and distributions across different subgroups, including sex and treatments. Statistical analysis was performed using the IBM SPSS Statistics 26 program. Scale variables were compared using the Independent Sample *t*-test, and nominal variables were compared using the Chi square test and Fisher’s exact test, when needed.

Due to the small sample size and uneven follow-up periods between patients, only short-term outcomes were assessed.

The Ethics Committee of the Sheba Medical Center approved this study, which was granted a waiver for informed consent due to the use of de-identified data.

## 3. Results

Our cohort consisted of 22 AHA patients, 50% of whom were females. Age at presentation ranged between 22 and 102 with a median age of 71 and an interquartile range (IQR) of 33.75–83.00. The full demographic data of our patients are presented in Table 1.

The suspected etiology for AHA was pregnancy in six patients (27.2%), malignancy in five patients (22.7%), and autoimmune disease in one patient who suffered from Bullous Pemphigus (0.045%). In the remaining 10 patients, the etiology was idiopathic.

All patients were diagnosed in a hospital setting. Three patients in our cohort were diagnosed with cancer during the AHA investigation. Two patients were diagnosed with hematological malignancies (CLL and Splenic Lymphoma), and one patient was diagnosed with renal cell carcinoma (RCC).

Symptoms at the patients’ presentation were versatile: 18 patients (81.8%) presented with spontaneous cutaneous hematomas, two patients (9%) had a subdural hematoma, and one patient (0.045%) suffered from a post-partum hemorrhage (PPH).

FVIII levels at presentation ranged from <1% to 7%. Inhibitor levels at presentation were between 1.1 BU and 343 BU, with a median of 18.5BU (IQR 5.5–29.75).

Only two patients (9%) required hemostatic treatment; of them, one had severe PPH, and the other had a subdural hematoma. Both patients were treated with rFVII (NovoSeven^®^ RT, Novo Nordisk, Bagsværd, Denmark).

One patient received human recombinant FVIII, and dosing was tailored according to repeated FVIII activity measurements, starting with 70–80 IU/kg, and repeated thrice daily, and later tapered off in response to inhibitor decline and a gradual increase in FVIII levels.

Following the diagnosis, most patients received immunosuppressive therapy (IST). Almost all patients (20/22) were treated initially with corticosteroids, and 10 patients (45%) required no further IST. Of the two patients not receiving immunosuppressive therapy, one was lost to follow-up, and the other declined treatment. Eight patients (36%) were further treated with Rituximab, five of whom (75%) suffered from post-partum AHA. Four patients (13.6%) were treated with Cytoxan, and one patient (0.045%) was treated with Emicizumab. Two patients (0.09%) collected from the early years of the cohort were also treated with Intra-venous Immunoglobulins (IVIg).

The patient requiring treatment with Emicizumab was a 26-year-old female, six months post-partum, who presented with lower-limb hematomas, and was diagnosed with AHA at another facility. Despite initial treatment with prednisone, azathioprine, and FFP, she developed an infected internal iliac muscle hematoma with a decrease in her hemoglobin levels to 7.3 g/dL from 12 g/dL, requiring transfer to our center. Upon admission, her aPTT was >150 s, her FVIII level was <1%, and her FVIII inhibitor level was 13.6 BU. Initial management included the administration of antibiotics, rFVII for hemostasis, and Rituximab for IST, leading to discharge with an FVIII level of 8%. The hematoma was resolved. Despite ongoing IST, she developed a new intramuscular bleed in her right calf, necessitating readmission. During the second admission, her hemoglobin level had dropped from 12.38 mg/dL to 9.2 mg/dL, her FVIII level to <1%, and her inhibitor level increased to 2.7 BU. In light of these findings, treatment with Emicizumab was initiated. Following the loading dose, her hemoglobin levels stabilized, and chromogenic FVIII levels increased to 19%.

The treatment strategies used and outcomes are delineated in Table 2.

**Figure 1:**
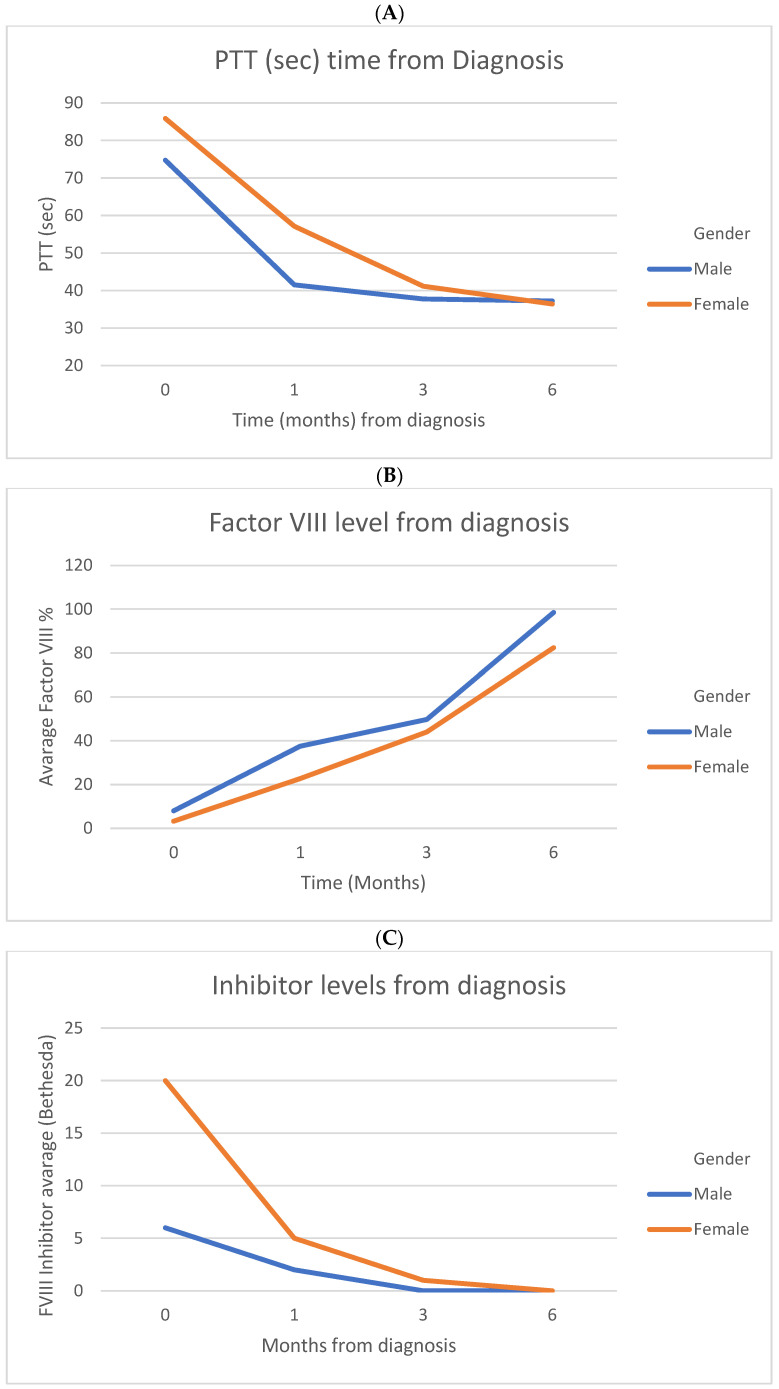
(**A**) Prothrombin time (PTT) in seconds over time (months) from diagnosis in male and female patients with acquired hemophilia A. Initial PTT levels were elevated in both genders, with a progressive decrease observed during follow-up, reaching comparable levels by six months post-diagnosis. (**B**) Average factor VIII levels (%) over time (months) from diagnosis in male and female patients with acquired hemophilia A. Both genders showed progressive increases in factor VIII levels during follow-up, with males demonstrating slightly higher levels by six months. (**C**) Average factor VIII inhibitor levels (%) over time (months) from diagnosis in male and female patients with acquired hemophilia A. Both genders showed progressive decrease in factor VIII inhibitor levels during follow-up period.

FVIII inhibitor was eradicated in 14/22 patients after a median of 3 months. Three patients (13.6%) failed to achieve full remission and reached FVIII levels of about 20%. Five patients were lost to follow-up. The dynamics of laboratory assays, including values of PTT, FVIII, and FVIII inhibitor, are shown in Figure 1 (panels A, B, and C, respectively). Additional information regarding laboratory values, treatment strategies, and outcomes for individual cohort patients can be found in Table S1 in the Supplementary Materials.

**Figure 2:**
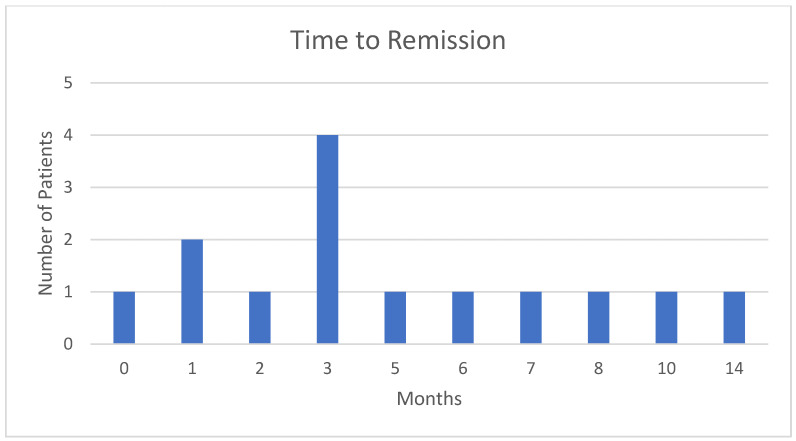
Time to AHA remission. Most patients (10/14) with remission achieved it in a period of up to 6 months from diagnosis.

Figure 2 demonstrates the time to AHA remission in our cohort. Most patients (10/14) with remission achieved it in a period of up to 6 months from diagnosis. One patient achieved remission after a longer period of 14 months despite being treated with IST, which included both corticosteroids and Rituximab.

One patient who had a pregnancy-related AHA and refused treatment had spontaneous remission after 3 months. She later underwent another pregnancy with no recurrence of the AHA.

Most patients did not report side effects from the treatment. One patient was suffering from corticosteroid side effects, including elevated blood pressure, weight gain, and moon face, and therefore the treatment was replaced with Rituximab. An 87-year-old woman suffered a fall and a hip fracture eight months after beginning corticosteroid treatment.

There were no cases of thrombosis in our cohort.

## 4. Discussion

In this single-center case series, we see a similar demographic distribution of patients and etiologies to that described in previous studies [1]. We did not find any statistically significant difference between male and female baseline characteristics, apart from the obvious peri-partum AHA etiology among women.

Initial immunosuppressive therapy was corticosteroid therapy as recommended in the literature. Although current guidelines suggest adding an additional IST such as Rituximab or Cytoxan, depending on the inhibitor level, in this cohort, the decision to add another immunosuppressive agent was attributed to the development of corticosteroid side effects (n = 2), an insufficient increase in FVIII levels (n = 6), or an insufficient decrease in FVIII inhibitor levels (n = 4). The use of Rituximab was more common in women who had AHA attributed to pregnancy, perhaps because it has less cytotoxic side effects as compared to Cytoxan. A previous nationwide study of the incidence, treatment, and outcomes of AHA in patients, conducted in Finland, demonstrated that treatment with Rituximab was more common in younger patients [12]. A different case series, comparing IST with Cyclophosphamide versus Rituximab, showed similar efficacy and safety, yet in patients with poor prognosis, Cyclophosphamide seems preferable, since significantly more remissions were observed with Cyclophosphamide [13].

One patient in our cohort, an extremely elderly woman—102 years old at the time of diagnosis, had cancer-associated AHA. She refused any treatment for her primary illness and went into remission of AHA 2 months after beginning treatment with steroids. No relapse was noted in the year following, despite steroid tapering and cession. This case raises questions about spontaneous remission in AHA. One patient in our cohort, a women post-partum, refused treatment and went into spontaneous remission. It has been shown in the past that patients with AHA can experience spontaneous remission, but some patients may suffer fatal bleeds [14]; regarding patients with post-partum AHA, it has been argued before that the natural history of acquired hemophilia post-partum is independent of immunosuppressive treatment, and that a spontaneous disappearance of the inhibitor against factor VIII occurs in the majority of cases [7,15]. Interestingly, despite bleeding manifestations (see Table 1) hemostatic treatment (either rFVIIa or high-dose FVIII) was only provided in three cases of severe bleeding, which led to a decrease in hemoglobin levels or was life-threatening.

Whilst thrombosis may be a common complication of AHA, associated with comorbidities of patients as well as with hypercoagulable states following bypass agent administration [16], no thrombosis was noted in our cohort. This could stem from the fact that only two patients received rFVIIa therapy for relatively short periods (1–2 days only) in order to control bleeding manifestations.

Current treatment recommendations for AHA rely on IST, which is effective but may cause severe side effects, like infections and a decrease in bone density [17]. As described above, one elderly woman in our cohort suffered a hip fracture during steroid treatment. Some other steroid-associated side effects documented in our cohort were hypertension and insomnia, all of which were treatable and reversible once IST was stopped. 

Nonetheless, as IST is not benign, any effective “IST sparing” therapies may potentially increase patients’ safety and improve AHA outcomes. Treatment with Emicizumab in this fragile population might be a game changer in the management of AHA [18]. Several studies have shown Emicizumab’s efficacy in decreasing bleeds and simultaneously decreasing the side effects of IST and bypassing agents. Tiede et al. recently published the findings of a phase 2 trial showing that the administration of Emicizumab to AHA patients can prevent bleeding events without co-administration of IST, thus reducing the number of thromboembolic events, severe infections, and fatalities [19]. In another study, Shima et al. published the results of an ongoing prospective, multicenter, open-label phase III study evaluating the efficacy, safety, and pharmacokinetics of Emicizumab, demonstrating that plasma Emicizumab concentration reached a steady state within a week; similarly to treatment results in congenital hemophilia, no major bleeds occurred in any patient. Neither death due to bleeding or infection nor any study treatment-related serious adverse event were reported [20].

In our cohort, only one woman was treated with Emicizumab. She was 24 years old, post-partum, and suffered severe cutaneous and muscular bleeds, and kept bleeding despite treatment with IST and bypassing agents. As social circumstances prevented the patient from regular clinic follow-up and there was a need to find a quick, safe, and efficient solution for the bleeding, Emicizumab was initiated shortly after her diagnosis. After a loading dose of Emicizumab, as suggestd by consensus recommendations, of 6 mg/kg body weight on day 1 and 3 mg/kg body weight on day 2 were used for rapid bleeding prophylaxis [21], and the bleeding stopped. No additional agent was used during this treatment. Unfortunately, the patient was lost for further follow-up later.

The recent consensus recommendations regarding AHA recommend Emicizumab as first- or second-line care for AHA, depending on the patient’s clinical manifestations and bleeding diathesis [21].

Another young patient with post-partum AHA after her first pregnancy was treated with IST with steroids and, due to a slow rise in FVIII levels, was further treated with Rituximab. She managed to achieve FVIII levels of about 20% and had no more spontaneous hematomas. She asked about timing another pregnancy, and after a review of the literature, was advised to continue birth control until she reached normal levels of FVIII. No previous description of pregnancy in a woman failing to achieve remission was found in the literature review. The risk of antibody transfer to the fetus has substantial consequences. The question of consecutive pregnancies despite failing to achieve full remission requires further research.

Three patients in this cohort were diagnosed with cancer during the investigation of AHA etiology. In most cases, the diagnosis of AHA is made after the cancer is diagnosed, but a 2012 review of 58 articles on the subject revealed seven case reports in which AHA diagnosis was made 2–7 months prior to cancer diagnosis [4]. This emphasizes the importance of cancer screening in patients presenting with idiopathic AHA.

Recurrence rates were in accordance with the literature [12]: similar between men and women. The trigger for recurrence was an invasive procedure in one case, and an aggravation of the disease burden in another.

This study has several limitations. This is a retrospective cohort with 7/22 patients who were lost to follow-up after initial admission. The significant proportion of patients lost to follow-up in our cohort makes the assessment of long-term outcomes difficult.

Due to the rarity of AHA, treatment strategies were not uniform and were changed with time. We believe that future studies with a prolonged follow-up period involving a larger number of patients can help overcome the limitations of this study, and provide more accurate information regarding suitable treatment strategies for this patient population. Nonetheless, our cohort confirms the known risk factors for AHA occurrence in old men and young females, highlighting the importance of cancer screening, as AHA presentation may precede cancer diagnosis, and shows the importance of IST-sparing treatment, such as hemostatic non-replacement therapies, e.g., Emicizumab. Future studies are required to better assess the chance of recurrence in specific populations, such as women of childbearing age undergoing consecutive pregnancies after full or partial remission.

## Figures and Tables

**Table 1:** Demographic information.

		Men	Women	*p*-Value
Age at presentation (years)		70.18	55.64	0.183
Past Medical History	Diabetes mellitus	4 (45.5%)	1 (10%)	0.94
	Hypertension	6 (54.5%)	4 (40%)	0.41
	CVA or TIA	1 (9.1%)	0	0.524
	Dementia	0	1 (10%)	0.476
	Atrial Fibrillation	1 (9.1%)	0	0.524
	Dyslipidemia	5 (45.5%)	2 (20%)	0.221
	Ischemic Heart Disease	2 (18.2%)	0	0.262
	Anemia	2 (18.2%)	1 (10%)	0.538
	Smoking	5 (45.5%)	1 (10%)	0.094
	Benign Prostatic Hyperplasia	2 (18.2%)	0	0.262
Suspected Etiology	Malignancy	5 (45.5%)	1 (9.1%)	0.74
	Pregnancy	0	6 (54.5%)	0.006
	Autoimmune Disease	1 (9.1%)	0	0.5
	Idiopathic	5 (45.5%)	4 (36.4%)	0.5
Clinical Manifestation	Cutaneous Hematoma	9 (81.8%)	11 (100%)	0.238
	Post-Partum Hemorrhage	0	1 (9.1%)	0.5
	Intra-Cranial Hemorrhage	2 (18.2%)	0	0.238
	Hematuria	2 (18.2%)	0	0.238

**Table 2:** Treatment strategies and outcomes.

		Men	Women	*p*-Value
Immunosuppressive Therapy	Corticosteroids	11 (100%)	9 (81.8%)	0.238
	Rituximab	3 (27.3%)	5 (45.5%)	0.330
	Cyclophosphamide	2 (18.2%)	2 (18.2%)	0.707
IVIg		2 (18.2%)	0	0.238
Emicizumab		0	1 (9.1%)	0.5
FVIII (Human Recombinant)		1 (9.1%)	0	0.5
rFVII		3 (27.3%)	1 (9.1%)	0.239
Remission (%)		6 (54.5%)	8 (72.7%)	0.238
Time to Remission (Months)		2.83	6.13	0.125
Relapse		2 (18.2%)	1 (9.1%)	0.538

## Supplementary Materials

The following supporting information can be downloaded at https://www.mdpi.com/article/10.3390/jcm14051597/s1, Table S1: Laboratory values, treatment strategies and outcomes for cohort patients.
