# Supplementary material for: Acquired Hemophilia—A Case Series and Review
Source: J Clin Med. 2025 Feb 26;14(5):1597. doi: 10.3390/jcm14051597 (PMC11901076; doi:10.3390/jcm14051597)
Supplement: Supplementary file 1 [file jcm-14-01597-s001.zip › jcm-3466671-supplementary.pdf]

Supplementary Table S1. Laboratory values, treatment strategies and outcomes for cohort patients

	Laboratory at presentation			Immunosuppressive therapy			Outcomes		
	PTT (sec)	FVIII (%)	FVIII Inhibitor (BU)	Non	Steroids	Additional IST	Follow up duration (months)	Recovered FVIII levels	Recurrence of bleeding
1	103	1	24		+		1		
2	74	3	16		+	+	6	+	
3	58	9	68		+		3	+	
4	66	4	56			+	1		
5	68	2	48		+		3	+	
6	46	2	16		+		6	+	
7	61	7	4		+	+	6	+	
8	90	0	20		+	+	6	+	
9	99	0	28		+	+	3		
10	47	28	1		+		6	+	+
11	104	0	44		+		3	+	
12	65	7	24		+	+	6	+	
13	150	0	14		+	+	3		
14	137	1	10		+		6	+	
15	90	0	4		+	+	6	+	
16	109	0	6		+	+	6	+	
17	61	5	3	+			3	+	
18	97	2	4		+	+	6	+	
19	102	0	18		+	+	3	+	+
20	84	1	19		+		6	+	
21	63	0	20		+		3	+	
22	109	0	35		+		3	+	
